# Case Report: Clinical and Imaging Characteristics of a Patient with Anti-flotillin Autoantibodies: Neuromyelitis Optica or Multiple Sclerosis?

**DOI:** 10.3389/fimmu.2021.808420

**Published:** 2021-12-23

**Authors:** Ke Shang, Chang Cheng, Chuan Qin, Jun Xiao, Gang Deng, Bi-Tao Bu, Sha-Bei Xu, Dai-Shi Tian

**Affiliations:** Department of Neurology, Tongji Hospital, Tongji Medical College, Huazhong University of Science and Technology, Wuhan, China

**Keywords:** demyelination, flotillin, neuromyelitis optica, multiple sclerosis, anti-flotillin autoantibody, case report

## Abstract

**Background:**

Demyelination diseases are complex puzzles that are not always straightforward to diagnose. Multiple sclerosis and neuromyelitis optica are two that are frequently encountered. Numerous autoantibodies newly discovered in recent years have significantly aided clinical reasoning and diagnosis in differentiating demyelination disorders. Here we report a case of demyelination disease with anti-flotillin autoantibodies positive, which is not common in past references.

**Case summary:**

The patient presented with characteristic neuromyelitis optica symptoms and had remission and relapse. But his images exhibited characteristics of both neuromyelitis optica spectrum illness and multiple sclerosis.

**Conclusion:**

This is the first case report describing the clinical course and imaging characteristics of demyelination illness associated with anti-flotillin autoantibodies. Although so far it appears to be a subtype of multiple sclerosis, there is still a potential that it is separate from MS and NMOSD.

## Introduction

Flotillins are considered to be scaffolding proteins of lipid rafts and are reported to participate in axon regeneration, neuronal differentiation, endocytosis, T-lymphocyte activation, membrane protein recruitment, insulin signaling, cell proliferation, and tumor progression (1, 2). As to flotillins’ role in diseases, they had been reported in Alzheimer’s disease (AD) (1, 3). Serum flotillin levels are significantly decreased in patients with AD and amyloid-positive MCI (Mild cognition impairment) patients compared with age-matched patients with VaD (Vascular dementia) and MCI patients without amyloid. The decrease in flotillin levels may result from reduced exosome secretion caused by Aβ42 oligomers. Hahn et al. reported the presence of autoantibodies against the flotillin-1/2 heterocomplex in the serum and CSF of patients with multiple sclerosis (4). Here, we first described the clinical and imaging characteristics of a patient with anti-flotillin autoantibodies in detail.

## Case Presentation

### Patient Presentation

In 2017, a 26-year-old man experienced a sudden reduction of vision in his left eye without eye movement pain or diplopia. Upon presentation to another hospital, he was diagnosed with optic neuritis and underwent treatment with high-dose intravenous methylprednisolone. Two weeks later, his left eye visual acuity returned to normal. In 2020, he was admitted to our hospital because of blurred vision that had been present in the left eye for 3 months and progressive lower limb weakness that had been present for 1 month. He did not exhibit other new symptoms. Cerebral and cervical magnetic resonance imaging in another hospital revealed multiple demyelinating lesions in the brainstem, bilateral insula, corpus callosum, left parietal lobe, and cervical cord (at the level of C3–C5). The patient had a history of 4–5 cigarettes per day for 8 years. He had no history of rheumatic disorders or other systemic disorders; he also had no history of mental or genetic disorders. His parents did not have a consanguineous relationship.

### Physical Examinations

Neurological examination revealed horizontal nystagmus and the presence of weakened light perception and limited abduction in the left eye. Muscle strength in the patient’s lower limbs was 80% of normal (4/5) under normal muscular tension. The patient’s tendon reflexes were brisk in both lower extremities, but normal in the upper limbs. He exhibited bilateral positive Babinski signs, as well as hemi-thermanalgesia immediately below the level of T8. His other physical findings were unremarkable.

### Laboratory Findings

Cerebrospinal fluid (CSF) examination revealed pressure of 0.59 kPa and nucleated cell count of 3*10^6^/L (reference: 0–8*10^6^/L). The patient’s immunoglobulin G index was 1.3 (reference: 0.00–0.70). CSF biochemical, immunological, microbiological, and virological tests showed no abnormalities. Complete blood count, biochemical test results, and findings concerning TPPA, HIV, HBV, HCV, tumor markers, thyroid function, and HbA1c were normal. Furthermore, ANA, RF, ANCA, and LA results were in normal ranges. Oligoclonal band and anti-flotillin-1/2 autoantibodies (1:1) were present in CSF but absent from serum; anti-AQP4, anti-MOG, and anti-MBP autoantibodies were absent from both CSF and serum.

### Imaging Data

Chest CT findings were normal. Bilateral optic nerves showed strong signals on T2WI with minimal gadolinium enhancement on T1WI. Spinal MRI revealed long-segment extensive lesions without enhancement, extending from C3 to C5 and from Th5 to Th10. Cerebral MRI showed abnormal long T1 and T2 signals at the bilateral frontal parietal lobe, centrum ovale, bilateral lateral ventricle, corpus callosum, hippocampus, and right cerebellar peduncles; these signals did not show mild enhancement or strong signals on DWI.

### Other Examinations

The corrected decimal visual acuity values were 0.08 and 0.1 in the patient’s left and left eyes, respectively. Fundus examination and OCT indicated retrobulbar neuritis in the left eye. Visual evoked potential analysis confirmed visual pathway impairment in both eyes. Brainstem auditory evoked potential analysis showed relatively low amplitudes with normal latency, which indicated conduction dysfunction in the peripheral segment of bilateral brainstem auditory pathways. Sensory-evoked potential analysis suggested a conduction dysfunction in the central segment of the bilateral lower limb sensory pathway with normal SEP of the upper limbs.

### Diagnosis, Treatment, and Follow-Up

**Figure 1:**
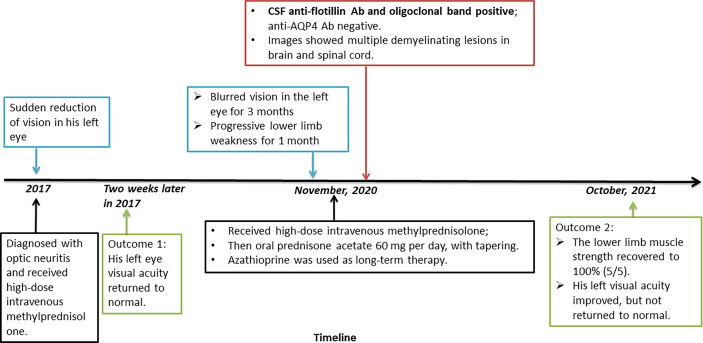
Timeline of the case report.

Based on the patient’s history, symptoms, and signs, including classical demyelination and evidence of anti-flotillin autoantibody, he was diagnosed with NMOSD. **Figure 1** was showcasing a timeline with relevant data from the episode of care. Therefore, he received high-dose intravenous methylprednisolone (initially 1 g per day, then tapering by half at 3-day intervals). He then received oral prednisone acetate 60 mg per day, with tapering. He also received immunosuppressants; tacrolimus was initially used, but azathioprine was subsequently used because the tacrolimus trough level was insufficient. The patient’s lower limb muscle strength recovered to 100% (5/5). His visual acuity in the left eye improved, but has not yet returned to normal. In the next face-to-face follow-up, radiological examinations including susceptibility weighted images, retest of CSF and serum autoantibodies, and thorough cognition assessment were scheduled.

## Discussion

Flotillins, also called reggies, are considered to be scaffolding proteins of lipid rafts and are generally used as marker proteins of lipid microdomains. They were initially identified as regeneration molecules that demonstrated upregulation in the regenerating axons of goldfish retinal ganglion cells following optic nerve lesion (5). There are two homologous proteins in flotillin family, flotillin-1 and flotillin-2. Flotillin-1 is expressed most abundantly in brain, heart, lung, and placenta, as well as hematopoietic cells; in contrast, flotillin-2 is expressed in all tissues; the expression of one is regulated by the other. These proteins are ubiquitous and highly conserved lipid raft scaffolding proteins (2). Flotillins do not span the membrane, but interact with other proteins on the other side of the membrane (6). Flotillins reportedly participate in axon regeneration, neuronal differentiation, endocytosis, T-lymphocyte activation, membrane protein recruitment, insulin signaling, cell proliferation, and tumor progression (1, 2). In neuronal cells, flotillins might modulate cadherin-mediated cell–cell adhesion, which is important for synapse organization and function, neuron regeneration, and dendritic spine morphogenesis (2). After reviewing basic functions of flotillins, we first went back to the clinical course of this patient.

**Figure 2:**
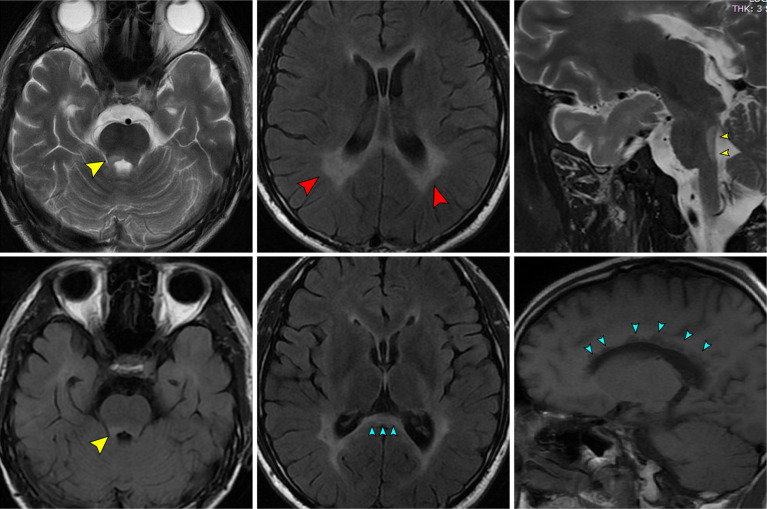
The patient’s MR images which mimicking NMOSD.

The patient’s first disease onset in 2017 comprised optic neuritis without limb weakness or numbness. Treatment with high-dose methylprednisolone provided rapid remission. It is unclear whether the patient received anti-AQP4 autoantibody detection at the time. In 2020, the patient presented with myelitis, including new symptoms such as lower limb weakness, and sensory disorder below the level of T8. The patient was initially misdiagnosed with NMOSD, on the basis of his disease beginning characteristics (neuritis and myelitis) and imaging findings. The international consensus diagnostic criteria for NMOSD were considered for this patient (7). Although he did not exhibit anti-AQP4, anti-MOG, or anti-MBP autoantibodies in the CSF or serum, he demonstrated two core clinical characteristics: acute optic neuritis and acute myelitis. Furthermore, he had some typical NMOSD lesions on cerebral MRI (**Figure 2**). Nearly symmetrical confluent deep white matter lesions were distributed around bilateral ventricles (red arrows in **Figure 2**); the corpus callosum (blue arrows in **Figure 2**) and associated periependymal brainstem (yellow arrows in **Figure 2**) were also involved.

**Figure 3:**
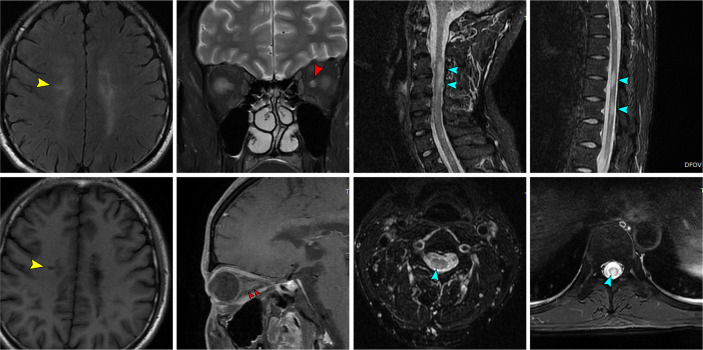
The patient’s MR images which mimicking MS.

However, a search of the literature revealed that anti-flotillin autoantibodies were mostly identified in reports of multiple sclerosis patients except for one recent case which reported anti-flotillin 1/2 autoantibody-associated atypical dementia (8). Furthermore, the patient’s optic nerve and spinal cord lesions did not clearly meet the diagnostic criteria for NMOSD (7). The T1-weighted gadolinium enhancing lesion in the patient’s optic nerve did not extend over more than half of the optic nerve length (red arrows in **Figure 3**); neither cervical nor thoracic lesions extended over three contiguous segments (blue arrows in **Figure 3**). Dawson’s fingers (i.e., elongated flame-shaped lesions perpendicular to the lateral ventricle wall) were observed on FLAIR images (yellow arrows in **Figure 3**); in light of the patient’s age, his brain displayed slight atrophy. The presence of Dawson’s fingers and brain atrophy are considered indicative of multiple sclerosis (9, 10). Consideration of the above posed a difficulty to the diagnosis of NMOSD.

In Hahn’s research (4), most of the anti-flotillin-1/2-positive patients had no evidence for a disorder other than MS. CSF analysis had revealed mild pleocytosis and/or CSF-specific oligoclonal band (OCB) in all patients. Thirteen (93%) patients were female. Nine (64%) patients had a history of optic neuritis. None of the patients displayed anti-AQP4 or anti-MOG antibodies. And none met the latest international consensus criteria for NMOSD and all demonstrated ‘red flag’ criteria according to that consensus (7). This study did not describe radiological signs of disseminated demyelination in detail, except for those described in the ‘red flag’ section, such as symmetrical confluent deep white matter lesions surrounding bilateral ventricles, lesions in the corpus callosum, and periependymal brainstem lesions, which were obvious in this case. These lesions, when paired with two key clinical characteristics: acute optic neuritis and acute myelitis, easily misled us into diagnosing NMOSD; nevertheless, a ‘red flag’ informed us of the need for a correction diagnosis of MS with anti-flotillin autoantibodies positive. Based on Hahn’s research (4) in a large cohort of pre-diagnosed MS patients, the flotillin-1/2 heterocomplex seems to be a B cell autoantigen in a subset of about 1–2% of MS patients. We hadn’t found studies or cases reporting anti-flotillin autoantibodies positive in Asian population. As more understanding of it and more patients diagnosed, we speculated that it would become a new subtype of MS or a disease distinctive from MS. Since flotillins had been reported as a possible new marker of AD (11, 12) and recently found in a patient of atypical dementia (8), besides classical symptoms and relapses, this patient’s cognition function requires further care during long-term follow-up.

Given a deep understanding of flotillins’ physiological functions, it’s not hard to infer why optic nerves, deep white matter and spinal cord are prone to be impaired by anti-flotillin autoantibodies. In early years, flotillins were found to be unregulated in retinal ganglion cells (RGCs) in fish (5) and the same in mammalian RGCs (13), which might explained vision loss. Munderloh et al. found that downregulation of flotillins triggered a clear reduction (up to 70%) of the number of regenerating axon in zebrafish and the knockdown of flotillins by flotillin-specific siRNAs restrained the axon regeneration (14), indicating that flotillins are indeed necessary for axon regeneration, which might illustrate the deep white matter and spinal cord lesions. Besides, flotillins had been reported to participate in hippocampal neurons differentiation. Downregulation of expression levels of flotillins in mammalian hippocampal neurons caused the neurons failed to differentiate (15). Langhorst et al. reported that expression of a trans-negative flotillin-2 deletion mutant, which interfered the oligomerization of flotillin, could inhibit insulin-like growth factor (IGF)-induced neurite outgrowth in N2a cells and impair differentiation of primary rat hippocampal neurons *in vitro* (16). These clues also reminded us to notice if there exists hippocampal injury and cognition impairment during long-term follow-up of this patient. Therefore, based on flotillins’ role in axon regeneration and RGCs, clinical symptoms and imaging characteristics of this patient were reasonable, despite the parallels and contrasts with NMOSD or MS.

This case remained a source of contention. To begin, the titer of anti-flotillin autoantibodies (1:1) was low, and this was the first time anti-flotillin autoantibodies had been found in the laboratory. To validate it, the laboratory conducted tests twice using cell-based assays (CBA) and enzyme-linked immunosorbent assays (ELISA). We addressed it in our department of neurology and determined that it was not a false positive based on the patient’s clinical presentations and a review of the literature. In due course, we will follow up on this case and reexamine his anti-flotillin autoantibodies in CSF. Second, long-term immunosuppressive medication requires additional observation. In our discussion, the diagnosis of MS and the subtype of relapse-remission MS (RRMS) are contentious. It is well established that disease modifying therapy is the first line of treatment for remission in RRMS. However, it has been noted that several disease-modifying medicines can exacerbate neuromyelitis optica (17–20). We picked tacrolimus and converted it to azathioprine after extensive consideration. As of last month, there has been no relapse attack.

Finally, given that flotillin-1/2 heterocomplexes have been detected on the cell surfaces of neural cells *in vivo*, mostly present on the intracellular side or temporarily on the extracellular side, corresponding autoantibodies could be pathogenic and contribute to demyelination. Demyelination disorders induced by anti-flotillin autoantibodies need further investigation and might constitute independent clinical entities that are distinct from NMOSD and MS.

## Data Availability Statement

The original contributions presented in the study are included in the article/supplementary material. Further inquiries can be directed to the corresponding authors.

## Ethics Statement

Written informed consent was obtained from the patient for the publication of any potentially identifiable images or data included in the article.

## Author Contributions

KS and CC equally contributed to the management of the patient and writing of the manuscript. CQ, JX, and GD contributed to a search of relevant references. B-TB raised meaningful opinions on the patient’s image features. S-BX and D-ST equally participated in decision of the patient’s long-term therapy and the whole design. All authors contributed to the article and approved the submitted version.

## Conflict of Interest

The authors declare that the research was conducted in the absence of any commercial or financial relationships that could be construed as a potential conflict of interest.

## Publisher’s Note

All claims expressed in this article are solely those of the authors and do not necessarily represent those of their affiliated organizations, or those of the publisher, the editors and the reviewers. Any product that may be evaluated in this article, or claim that may be made by its manufacturer, is not guaranteed or endorsed by the publisher.
